# Notes and News

**Published:** 1901-10-19

**Authors:** 


### Notes and News.

We have received several communications in reference to an article concerning the Boer Concentration Camps which appeared in our issue of October 5th. We are afraid that the writer of the article in question made an error in regard to the mortality in these camps, and we regret that, in consequence of death-rates being usually calculated per annum, we did not detect the mistake. The fact appears to be that the death-rate recorded referred only to one month, and the deductions drawn from it are erroneous.

A conviction by the Lynn Bench of magistrates this week serves as a striking example of what might frequently happen if any laxity of food inspection were to be encouraged by Dr. Koch's views on the intercommunicability of human bovine tuberculosis. A fellmonger received for purposes of his trade the carcase of a cow badly diseased with tuberculosis. Instead of using the carcase in a legitimate way of trade, he resold it for £9 to an inexperienced meat-buyer, whose employer, a widow, innocently exposed the meat for sale. For her unwitting share in the offence the woman was fined a small sum, but the Bench marked their sense of the gravity of the original vendor's misdoing by imposing a total penalty of £21.

Professor Virchow's eightieth birthday celebrations took place in Berlin on October 13th. The occasion will ever be remarkable in the history of medicine, for seldom have so many brilliant intellects assembled together or sent their tribute to honour a medical man. A marble bust of the Professor was presented to the Virchow Institute, and a banquet was held in his honour, during which the various foreign delegates delivered addresses. Lord Lister and Sir Felix Lemon represented England. Professor Virchow spoke at both ceremonies, and all were astonished at the clearness and vigour of his delivery. The German Emperor has conferred the Great Gold Medal for Science upon Dr. Virchow, and at the same time wrote him a letter of congratulation, which was read at the banquet.

The Nord Deutscher-Lloyd steamers are not now calling at Naples, owing to the presence of the plague there. They are embarking and landing passengers and mails at Genoa for the present.

The new Pathological Laboratory was opened at Oxford last Saturday. Sir William Church delivered an address on the occasion, as did also Professor Woodhead, of Cambridge. The laboratory has been erected at a cost of £10,000.

Mr. Lynn Thomas, F.R.C.S., surgeon to the Cardiff Infirmary, who had charge of the Welsh Hospital in South Africa after the death of Professor Hughes and Mr. Tom Jones, has been made a Companion of the Order of the Bath in recognition of his valuable services.

Major Thoyts, chairman of the Royal Berkshire Hospital, has been elected president of the institution in the place of the late Lord Wantage. The hospital is to undergo considerable alteration and improvement at an estimated cost of £16,000. Half this sum is to be provided by donations and half is to come from capital.

The Committee of Management of the North London Hospital for Consumption having asked five well-known hospital architects to compete for the erection of their proposed Country Branch and Convalescent Home at Northwood, Middlesex, have selected the design submitted by Mr. Frederick Wheeler, F.R.I.B.A., of 6 Staple Inn, W.C.

The latest aspect of the small pox in London is notable, not so much for the number of the cases as the area over which they are spread. Facilities of intercommunication add to the difficulty of localising and rapidly stamping out so infectious a disease as small-pox in the metropolis. The argument in favour of vaccination is very much strengthened by the fact.

The South Wales branch of the National Society for the Prevention of Consumption is carrying on a very active crusade. A Conference has just been held at Cardiff, very fully attended by the medical men and other influential people of the district, when much interest in the question was awakened, and a good deal of useful knowledge was disseminated.

The London County Council have made new bye-laws regulating the hours during which offensive matter may be moved through the public streets, and as to the receptacles in which it may be removed, so as to minimise any annoyance to the public. The hours are very liberal, seeing that they extend to noon during the winter months and to 10 a.m. in summer.

One of the Accident Assurance Societies has done a smart stroke of business. It has extended the scope of its policies to cover an attack of small-pox. After all there is nothing very misleading in describing small pox as a dangerous accident that may befall anyone. And at the same time we prophesy the society will lose nothing by its liberality —if it can induce its clients to be vaccinated!

